# Morte por Covid-19 em Indivíduos com Hipertensão e Sobrepeso: Diabetes pode ser Fator de Risco?

**DOI:** 10.36660/abc.20230332

**Published:** 2023-10-20

**Authors:** Nayara Ribeiro Máximo de Almeida, Brenda dos Santos Teixeira, Breno Rennan de Souza Carvalho, Egidio Bezerra da Silva, Maria Alice Leitão Araújo de Melo, Júlio Martinez Santos, Johnnatas Mikael Lopes

**Affiliations:** 1 Universidade Federal do Vale do São Francisco Paulo Afonso BA Brasil Universidade Federal do Vale do São Francisco (UNIVASF) – Medicina, Paulo Afonso , BA – Brasil; 2 UNINASSAU Recife PE Brasil Centro Universitário Maurício de Nassau ( UNINASSAU ), Recife , PE – Brasil

**Keywords:** COVID-19, Obesidade, Hipertensão, Morte, Estudos de Coortes

Em dezembro de 2019 na China, a infecção pelo vírus SARS-CoV-2 evoluiu rapidamente para uma pandemia. A proteína S do vírus SARS-CoV-2 liga-se à enzima conversora de angiotensina 2 (ECA 2), que faz parte do sistema renina-angiotensina-aldosterona (SRAA), para servir como um receptor de entrada celular. Esses receptores são amplamente expressos no coração, intestinos, rins, pâncreas e trato respiratório. ^1^

O SRAA é uma via de sinalização que atua como um regulador homeostático da função vascular, responsável pelo controle da pressão arterial. A ECA 2 é uma enzima que catalisa a clivagem da Angiotensina II em Angiotensina (1-7), Angiotensina I em Angiotensina (1-9) e participa da hidrólise de outros peptídeos. Dessa forma, os medicamentos Inibidores da Enzima Conversora de Angiotensina (IECA) e os Bloqueadores do Receptor AT1 da Angiotensina 2 (BRA) vem sendo bastante estudados pela possibilidade de aumentarem a expressão da ECA 2. ^2^

Vale salientar o papel central da ligação entre a proteína S do SARS-Co-2 e a ECA 2, como método promotor da entrada celular pelo vírus. Além da significativa expressão da ECA 2 na mucosa do trato respiratório, mesmo que em menor quantidade quando comparada a outros tecidos. ^3^ Também foi demonstrado que independentemente do sexo e etiologia, a utilização a longo prazo de IECA e de BRA não promove um aumento da expressão da ECA 2 na mucosa do trato respiratório. ^4^

O estudo de Sham et al., ^5^ publicado neste periódico v. 120, n. 4, aborda uma temática de extrema importância para o manejo de pacientes hipertensos e obesos em situações de infecção por COVID-19, por meio da correlação entre os antagonistas do sistema renina-angiotensina-aldosterona e os desfechos desfavoráveis da COVID-19. Os autores, talentosamente, desenvolveram um desenho de coorte retrospectiva, onde se mostrou a ordem cronológica das variáveis independentes e o desfecho.

Entretanto, observamos que os desfechos investigados possuem alta incidência, com exceção do ECMO. Isso implica na inadequação do odds ratio como medida de associação/predição, pois cria intervalos de confiança superdimensionados, produzindo, portanto, relações não significativas no modelo ajustado. ^6 , 7^ Destarte, essa situação pode ser evidenciada através das variáveis, diabetes e IMC, presentes no referido estudo.

A implicação nos resultados da investigação de Sham et al. ^5^ acarreta impactos nas revisões sistemáticas com metanálise que utilizam as estimativas intervalares e pontuais para efeito de intervenções. ^7^ À vista disso, há a possibilidade de evidências equivocadas serem aplicadas nas tomadas de decisões clínicas.

Mediante o exposto, sugerimos que esta análise seja realizada a partir da regressão de Poisson, a qual estima uma medida de efeito chamada risco relativo. ^6 , 7^ Tal regressão, apresenta valores pontuais e intervalares mais robustos, o que propicia evidenciar efeitos não detectados pela inflação dos intervalos de confiança criados pela regressão logística binária. Por fim, também sugerimos a apresentação dos valores brutos da medida de associação para melhor interpretação do modelo explicativo.
