# Supplementary material for: Morte por Covid-19 em Indivíduos com Hipertensão e Sobrepeso: Diabetes pode ser Fator de Risco?
Source: Arq Bras Cardiol. 2023 Oct 20;120(10):e20230332. [Article in Portuguese] doi: 10.36660/abc.20230332 (PMC10593385; doi:10.36660/abc.20230332)
Supplement: Translation

# Translation: Death by Covid-19 in Individuals with Hypertension and Overweight: Can Diabetes be a Risk Factor?

**Authors:** Nayara Ribeiro Máximo de Almeida, Brenda dos Santos Teixeira, Breno Rennan de Souza Carvalho, Egidio Bezerra da Silva, Maria Alice Leitão Araújo de Melo, Júlio Martinez Santos, Johnnatas Mikael Lopes

**Affiliations:** 1 Brazil Universidade Federal do Vale do São Francisco (UNIVASF) – Medicina, Paulo Afonso, BA – Brazil; 2 Brazil Centro Universitário Maurício de Nassau (UNINASSAU), Recife, PE – Brazil

**Keywords:** COVID-19, Obesity, Hypertension, Death, Cohort Studies

In China, December 2019, the SARS-CoV-2 virus infection rapidly evolved into a pandemic. The S protein from the SARS-CoV-2 virus binds to angiotensin-converting enzyme 2 (ACE 2), which is part of the renin-angiotensin-aldosterone system (RAAS), to serve as a cell-entry receptor. These receptors are widely expressed in the heart, intestines, kidneys, pancreas and respiratory tract. ^1^

The RAAS is a signaling pathway that acts as a homeostatic regulator of vascular function, responsible for blood pressure control. ACE 2 is an enzyme that catalyzes the cleavage of Angiotensin II into Angiotensin (1-7), Angiotensin I into Angiotensin (1-9) and participates in the hydrolysis of other peptides. Thus, Angiotensin-Converting Enzyme Inhibitors (ACE) and Angiotensin 2 AT1 Receptor Blockers (ARB) have been extensively studied due to the possibility of increasing ACE expression 2. ^2^

It is worth emphasizing the central role of the binding between the protein S of SARS-Co-2 and ACE 2, as a method to promote cell entry by the virus. In addition to the significant expression of ACE 2 in the respiratory tract mucosa, even if in smaller quantities when compared to other tissues. ^3^ It has also been shown that, regardless of gender and etiology, the long-term use of ACE inhibitors and ARBs does not promote an increase in ACE 2 expression in the respiratory tract mucosa. ^4^

The study by Sham et al., ^5^ published in this journal v. 120, no. 4, addresses a topic of extreme importance for the management of hypertensive and obese patients in situations of COVID-19 infection, through the correlation between antagonists of the renin-angiotensin-aldosterone system and the unfavorable outcomes of COVID-19. The authors, talentedly, developed a retrospective cohort design, where the chronological order of the independent variables and the outcome were shown.

However, we observed that the investigated outcomes have a high incidence, with the exception of ECMO. This implies that the odds ratio is inadequate as a measure of association/prediction, as it creates oversized confidence intervals, thus producing non-significant relationships in the adjusted model. ^6 , 7^ Thus, this situation can be evidenced through the variables, diabetes and BMI, present in that study.

The implication in the results of the investigation by Sham et al. ^5^ entails impacts on systematic reviews with meta-analysis that use interval and point estimates for the effects of interventions. ^7^ In view of this, there is the possibility of erroneous evidence being applied in clinical decision-making.

In view of the above, we suggest that this analysis be performed using Poisson regression, which estimates an effect measure called relative risk. ^6 , 7^ Such regression presents more robust punctual and interval values, which allows to evidence effects not detected by the inflation of the confidence intervals created by the binary logistic regression. Finally, we also suggest presenting the raw values of the association measure for a better interpretation of the explanatory model.
